# Risk of heart failure in ambulatory resistant hypertension: a meta-analysis of observational studies

**DOI:** 10.1038/s41440-024-01632-8

**Published:** 2024-03-14

**Authors:** Francesca Coccina, Gil F. Salles, José R. Banegas, Ramón C. Hermida, José M. Bastos, Claudia R. L. Cardoso, Guilherme C. Salles, Mercedes Sánchez-Martínez, Artemio Mojón, José R. Fernández, Carlos Costa, Simão Carvalho, Joao Faia, Sante D. Pierdomenico

**Affiliations:** 1 Department of Innovative Technologies in Medicine & Dentistry, University “Gabriele d’Annunzio”, Chieti-Pescara, Chieti, Italy; 2 Department of Internal Medicine, School of Medicine, Universidade Federal do Rio de Janeiro, Rio de Janeiro, Brazil; 3 Department of Preventive Medicine and Public Health, Universidad Autónoma de Madrid and CIBERESP, Madrid, Spain; 4 Bioengineering & Chronobiology Laboratories, Atlantic Research Center for Telecommunication Technologies (atlanTTic), Universidade de Vigo, Vigo, Spain; 5 Bioengineering & Chronobiology Research Group, Galicia Sur Health Research Institute (IIS Galicia Sur), SERGAS-UVIGO, Vigo, Spain; 6 School of Health Sciences and Institute of Biomedicine-iBiMED, University of Aveiro, Aveiro, Portugal; 7 Deparment of Civil Engineering, Polytechnic School, Universidade Federal do Rio de Janeiro, Rio de Janeiro, Brazil; 8 Department of Health Science, Universidad Católica Santa Teresa de Jesús de Ávila, Ávila, Spain; 9 Cardology Department of Centro Hospitalar Baixo Vouga, Aveiro, Portugal

**Keywords:** Ambulatory blood pressure, Heart failure, Hypertension, Resistant hypertension

## Abstract

The impact of ambulatory resistant hypertension (ARH) on the occurrence of heart failure (HF) is not yet completely known. We performed for the first time a meta-analysis, by using published data or available data from published databases, on the risk of HF in ARH. Patients with ARH (24-h BP ≥ 130/80 mmHg during treatment with ≥3 drugs) were compared with those with controlled hypertension (CH, clinic BP < 140/90 mmHg and 24-h BP < 130/80 mmHg regardless of the number of drugs used), white coat uncontrolled resistant hypertension (WCURH, clinic BP ≥ 140/90 mmHg and 24-h BP < 130/80 mmHg in treated patients) and ambulatory nonresistant hypertension (ANRH, 24-h BP ≥ 130/80 mmHg during therapy with ≤2 drugs). We identified six studies/databases including 21,365 patients who experienced 692 HF events. When ARH was compared with CH, WCURH, or ANRH, the overall adjusted hazard ratio for HF was 2.32 (95% confidence interval (CI) 1.45–3.72), 1.72 (95% CI 1.36–2.17), and 2.11 (95% CI 1.40–3.17), respectively, (all *P* < 0.001). For some comparisons a moderate heterogeneity was found. Though we did not find variables that could explain the heterogeneity, sensitivity analyses demonstrated that none of the studies had a significant influential effect on the overall estimate. When we evaluated the potential presence of publication bias and small-study effect and adjusted for missing studies identified by Duval and Tweedie’s method the estimates were slightly lower but remained significant. This meta-analysis shows that treated hypertensive patients with ARH are at approximately twice the risk of developing HF than other ambulatory BP phenotypes.

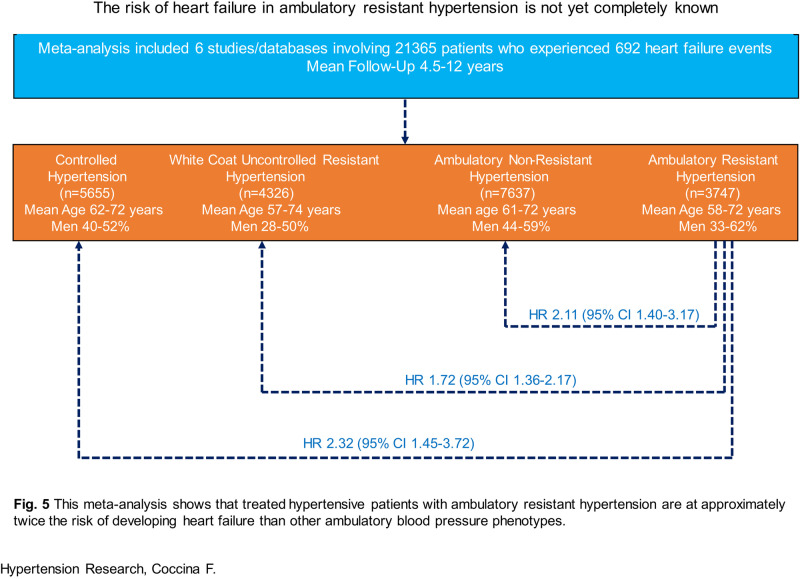

## Introduction

Ambulatory resistant hypertension (ARH) or true resistant hypertension is defined as high blood pressure (BP) in the clinic, despite use of three or more antihypertensive drugs, which is confirmed by ambulatory BP monitoring [1]. It may be present in more than 10% of treated hypertensive patients [2–4], and its frequency further increases in the elderly [4]. Diagnostic work-up and updated therapeutic strategy for this condition have recently been reported [1].

Various studies [5–19] have evaluated the risk profile in ARH and have shown that it is associated with substantial increased risk of cardiovascular events when compared to other out-of-office BP phenotypes. Usually, all major cardiovascular outcomes have been analyzed together in the evaluation of the prognostic impact of ARH [5–19]. Recently, to the best of our knowledge, only two studies [20, 21] have specifically reported a higher risk of heart failure (HF) in ARH. Thus, the impact of ARH on HF is not yet completely known.

HF is a relevant public health burden characterized by high mortality, hospitalizations, and re-hospitalizations rates and costs [22, 23]. In Western Countries, the prevalence of HF has been reported to be higher than 10% in elderly patients [23]. Hypertension is one of the most important causes of HF [24]. Indeed, it has been shown that its population attributable risk for HF is as high as that of coronary artery disease [25].

The prevalence of both ARH [1–4] and HF [22, 23] is progressively increasing and new drug classes have proven effective in preventing HF [26–29] and further reducing BP [26–31].

In this scenario, the aim of the present study was to perform a meta-analysis, by using published data on the specific topic of the association between ARH and HF and data available from published databases on the relationship between ARH and cardiovascular outcome, in the attempt to give a broader information about the risk of HF in ARH that could be of clinical relevance in future perspective. For this purpose, patients with ARH were compared with those exhibiting the other ambulatory on-treatment BP phenotypes.

## Methods

### Search strategy and selection criteria

This study was performed in accordance with the recommendations of the Meta-analysis of Observational Studies in Epidemiology group [32]. We conducted a literature search through PubMed, Web of Science, and Cochrane Library for articles evaluating the occurrence of HF in patients with ARH in comparison with other ambulatory BP phenotypes up to September 2023. The terms used to identify studies were “ambulatory resistant hypertension” or “true resistant hypertension” and “heart failure” or “cardiovascular outcome” or “cardiovascular events”. Two reviewers independently screened titles and abstracts to identify eligible studies or published databases from which to extrapolate the topic of this study. Reference lists of included articles were also examined for additional studies. Data were extracted from published manuscripts on the topic or requested to the investigators who published manuscripts dealing with the prognostic impact of ARH.

Inclusion criteria for entry in the meta-analysis were (1) full-text paper published in a peer-reviewed journal dealing with ARH and HF or dealing with the prognostic value of ARH from which potentially extrapolate data on the relationship between ARH and HF; (2) any language of publication; (3) study on adult population; (4) treated hypertensive population; (5) prospective study; (6) follow-up of at least 1 year; (7) use of ambulatory BP monitoring; (8) homogeneous definition of ambulatory BP phenotypes based on 24-h BP (from published data or on request for databases evaluating ARH); (9) assessment of the occurrence of HF in patients with ARH, defined as clinic BP < or ≥140/90 mmHg and 24-h BP ≥ 130/80 mmHg in patients taking three or more drugs (that is resistant masked uncontrolled hypertension and resistant sustained uncontrolled hypertension), compared to other ambulatory BP phenotypes, namely: (i) controlled hypertension (CH) defined as clinic BP < 140/90 mmHg and 24-h BP < 130/80 mmHg regardless of the number of drugs used; (ii) white coat uncontrolled resistant hypertension (WCURH) defined as clinic BP ≥ 140/90 mmHg and 24-h BP < 130/80 mmHg in patients receiving antihypertensive drugs; and (iii) ambulatory nonresistant hypertension (ANRH) defined as clinic BP  < or ≥140/90 mmHg and 24-h BP ≥ 130/80 mmHg in patients taking ≤2 drugs (that is non-resistant masked uncontrolled hypertension and non-resistant sustained uncontrolled hypertension), respectively; (10) comparisons could include all the aforesaid ambulatory BP phenotypes or only part of them, but necessarily including ARH; (11) availability of adjusted hazard ratio (HR) and 95% confidence interval (CI) between ARH and other ambulatory BP phenotypes.

### Study selection and data extraction

**Fig. 1:**
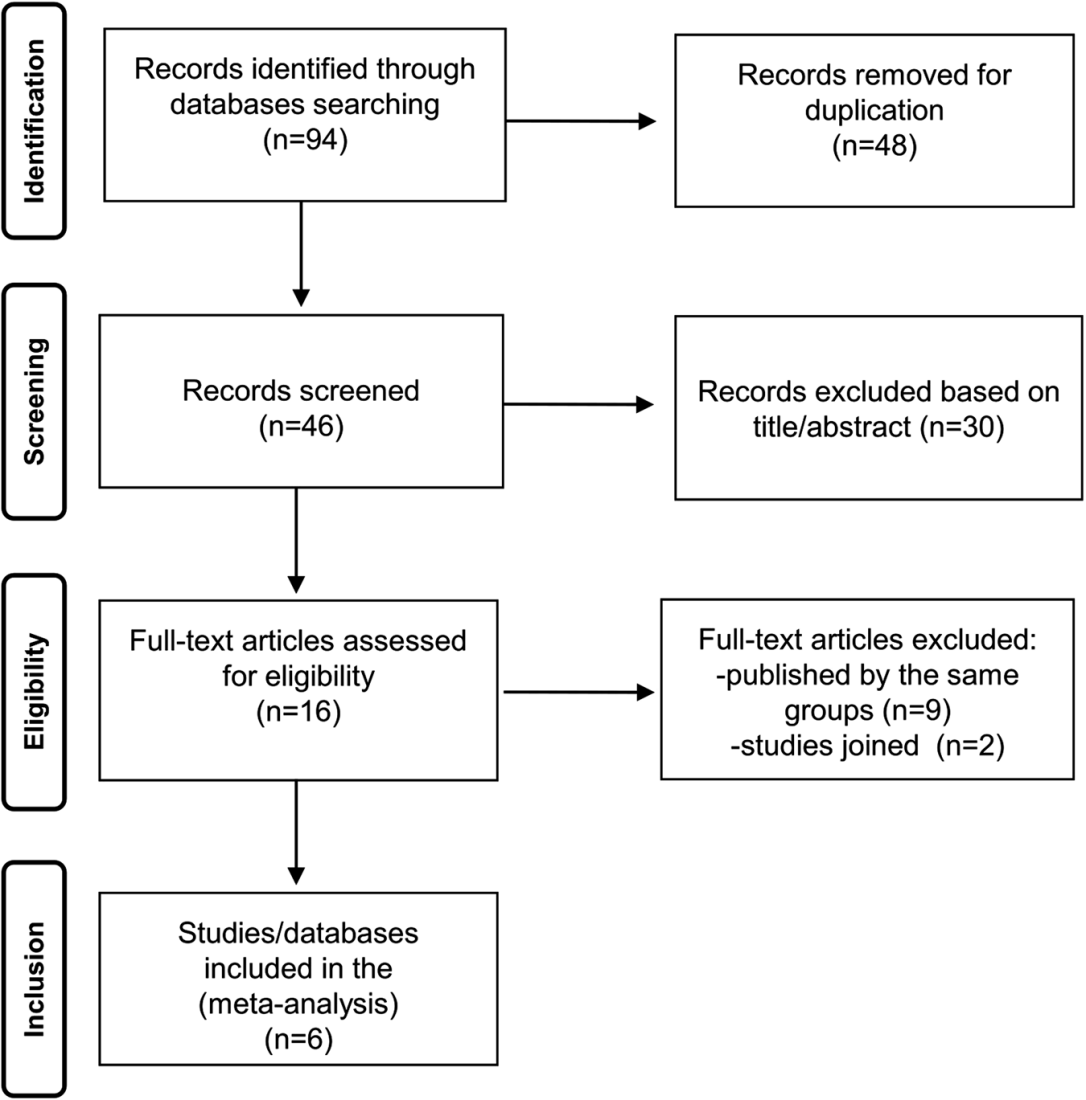
Flow chart showing selection of publications

By using the selected terms listed above, the first literature search identified 94 studies from revision of titles and abstract, 30 studies were excluded. Among the remaining 16 manuscripts, 2 studies [20, 21] were immediately eligible because dealing with the specific topic and other 14 studies [5–9, 11–19] could be eligible because dealing with the prognostic value of ARH in hypertensive patients in general. The authors (four study groups) of the aforesaid studies were contacted. They agreed to participate to the study and provided the estimated data [8, 13, 15, 16, 19]. References [5, 18] were published by the same authors of ref. [21] and were excluded; refs. [6, 7, 14, 17] were published by the same authors of ref. [16] and were excluded; refs. [11, 12] were published by the same authors of ref. [15] and were excluded; ref. [9] was excluded because of lack of prospective data on the topic. References [8, 19] were analyzed together. Finally, six studies/databases were selected for the meta-analysis (Fig. 1), that is, refs. [13, 15, 16, 20, 21], and refs. [8, 19] analyzed together. Two reviewers independently extracted relevant data from selected studies. The quality of included studies was assessed using the Newcastle–Ottawa scale [33]. This scale evaluates cohort studies based on (1) selection (representativeness of the exposed cohort, selection of the non-exposed cohort, ascertainment of exposure, demonstration that outcome of interest was not present at start of study; maximum 4 stars), (2) comparability (comparability of cohorts on the basis of the design or analysis; maximum 2 stars), and (3) outcome (assessment of outcome, follow-up length, adequacy of follow-up of cohorts; maximum 3 stars). The total maximum score can be 9.

### Statistical analysis

To address confounding, we used the adjusted HR and 95% CI of the individual studies to calculate the overall adjusted HR and 95% CI. We used the random effects model [34]. Tests of heterogeneity were performed using the Cochrane Q statistic and *I*^2^ statistic [35], and tau-squared statistics measured between-studies variance. Subgroup meta-analysis or meta-regression were also performed to analyze potential sources of heterogeneity [36]. Individual studies were removed one at a time to evaluate the influence of each study on the pooled estimate. A funnel plot, Begg and Mazumdar [37] rank correlation test, and Egger’s [38] regression test for funnel plot asymmetry were used to examine the likely presence of publication bias and small-study effect. Potential adjustment for missing studies was approached by Duval and Tweedie’s trim and fill method [39]. Statistical significance was defined as *P* < 0.05 (two-tailed tests). Analyses were done using the Comprehensive Meta-Analysis software version 2 (Biostat, Englewood, NJ).

## Results

The pooled population consisted of 21,365 patients who experienced 692 HF events and the mean follow-up ranged from 4.5 to 12 years (Table 1). All the studies defined ARH as 24-h BP ≥ 130/80 mmHg despite use of three or more antihypertensive drugs, including a diuretic unless discontinued because of side effects. Definition of HF in the studies is reported in Supplementary Table 1 and it was based on clinical and instrumental findings. Age, sex, body mass index, smoking habit, diabetes, cholesterol, previous events, renal function, and clinic and ambulatory systolic and diastolic BP are reported in Tables 2–4. In patients with ARH, mean age ranged from 58 to 72 years, prevalence of men from 33 to 62%, mean body mass index from 26 to 31 kg/m^2^, prevalence of diabetes from 24 to 51% and prevalence of previous events from 11 to 41%. A total of 24-h systolic BP was more than 20 mmHg higher in patients with ARH than in those with CH and WCURH and tended to be higher than in those with ANRH, and 24-h diastolic BP was about 10 mmHg higher in patients with ARH than in those with CH and WCURH. Other characteristics of the included studies/databases are reported in Supplementary Table 2. Four studies [8, 15, 16, 19, 20] evaluated general treated hypertensive patients and two evaluated elderly treated hypertensive patients [13, 21]. One study [20] assessed Japanese subjects, one study [16] Mixed Latinos, and four studies [8, 13, 15, 19, 21] Caucasians. All the studies used in multivariate analyses a set of covariates including main cardiovascular risk factors, and some of them used additional covariates. According to the Newcastle–Ottawa scale, all the included studies were of high quality (Supplementary Table 3).

**Table 1:** Participants, follow-up, and heart failure events of selected studies

Study	Patients per group				Total patients	Mean FU	HF events per group				Total HF events
	CH	WCURH	ANRH	ARH		(years)	CH	WCURH	ANRH	ARH	
JAMP Study [20]	2049	222	3147	421	5839	4.5	ND	ND	ND	ND	67
Chieti-Pescara Study [21]	153	153	307	132	745	8.4	10	9	39	24	82
Rio de Janeiro Study [16]	NE	672	NE	976	1648	7.7	NE	10	NE	27	37
Hygia Project Study [15]	2802	2821	3274	2047	10,944	5.5	95	99	103	130	427
ENRICA-Seniors Study [13]	522	169	532	68	1291	4.9	13	7	11	6	37
Aveiro Study [8, 19]	129	289	377	103	898	12	3	10	12	17	42

*ANRH* ambulatory nonresistant hypertension, *ARH* ambulatory (true) resistant hypertension, *CH* controlled hypertension, *ENRICA* Study on Nutrition and Cardiovascular Risk in Spain, *FU* follow-up, *HF* heart failure, *JAMP* Japan Ambulatory Blood Pressure Monitoring Prospective, *ND* not described, *NE* not evaluated, *WCURH* white coat uncontrolled resistant hypertension

**Table 2:** Age, sex, body mass index, and smoking habit of selected studies

Study	Age (mean)				Men (%)				Body mass index (mean)				Current smokers (%)			
	CH	WCURH	ANRH	ARH	CH	WCURH	ANRH	ARH	CH	WCURH	ANRH	ARH	CH	WCURH	ANRH	ARH
JAMP Study [20]	70	74	67	70	44	43	49	56	24	26	25	26	8	10	11	11
Chieti-Pescara Study [21]	71	72	71	72	40	36	44	40	27	28	27	28	14	6	9	10
Rio de Janeiro Study [16]	NE	63	NE	61	NE	28	NE	33	NE	30	NE	30	NE	6	NE	10
Hygia Project Study [15]	63	66	63	66	52	50	59	62	30	31	30	31	12	8	18	12
ENRICA-Seniors Study [13]	72	72	72	72	46	47	52	59	28	29	29	30	7	11	9	12
Aveiro Study [8, 19]	62	57	61	58	44	46	52	59	28	28	28	30	39	31	60	41^a^

Values up to 0.5 were rounded to the lower unit and those greater than 0.5 to the higher unit

*ANRH* ambulatory nonresistant hypertension, *ARH* ambulatory (true) resistant hypertension, *CH* controlled hypertension, *ENRICA* Study on Nutrition and Cardiovascular Risk in Spain, *JAMP* Japan Ambulatory Blood Pressure Monitoring Prospective, *NE* not evaluated, *WCURH* white coat uncontrolled resistant hypertension

^a^Current and past smokers

**Table 3:** Diabetes, cholesterol, previous events, and renal function of selected studies

Study	Diabetes (%)				Cholesterol (mean)				Previous events (%)				eGFR < 60 ml/min (%)			
	CH	WCURH	ANRH	ARH	CH	WCURH	ANRH	ARH	CH	WCURH	ANRH	ARH	CH	WCURH	ANRH	ARH
JAMP Study [20]	18	28	17	30	192	192	198	194^a^	14	17	11	22	NA	NA	NA	NA
Chieti-Pescara Study [21]	11	10	16	24	131	123	128	122^b^	7	14	9	11	39	50	42	54
Rio de Janeiro Study [16]	NE	54	NE	51	NE	202	NE	211^a^	NE	36	NE	41	NE	37	NE	47
Hygia Project Study [15]	26	29	26	47	194	197	200	193^a^	18	13	12	20	27	30	26	40
ENRICA-Seniors Study [13]	18	14	24	37	106	110	110	105^b^	19	17	16	22	6	10	7	15
Aveiro Study [8, 19]	28	27	27	48	37	34	16	68^c^	5	3	1	25	NA	NA	NA	NA

Values up to 0.5 were rounded to the lower unit and those greater than 0.5 to the higher unit

*ANRH* ambulatory nonresistant hypertension, *ARH* ambulatory (true) resistant hypertension, *CH* controlled hypertension, *ENRICA* Study on Nutrition and Cardiovascular Risk in Spain, *JAMP* Japan Ambulatory Blood Pressure Monitoring Prospective, *NA* not available, *NE* not evaluated, *WUCRH* white coat uncontrolled resistant hypertension

^a^Total cholesterol

^b^LDL cholesterol

^c^Percentage of patients with dyslipidemia (taking statins and/or with LDL cholesterol >130 mg/dl)

**Table 4:** Clinic and 24-h blood pressure of selected studies

Study	Clinic SBP (mean)				Clinic DBP (mean)				24-h SBP (mean)				24-h DBP (mean)			
	CH	WCURH	ANRH	ARH	CH	WCURH	ANRH	ARH	CH	WCURH	ANRH	ARH	CH	WCURH	ANRH	ARH
JAMP Study [20]	137	153	149	156	76	79	84	83	120	121	142	143	70	69	81	79
Chieti-Pescara Study [21]	130	153	159	160	77	87	89	88	118	122	140	143	69	70	77	76
Rio de Janeiro Study [16]	NE	158	NE	173	NE	86	NE	94	NE	118	NE	146	NE	67	NE	84
Hygia Project Study [15]	124	154	154	160	74	86	88	86	116	120	139	142	68	68	80	77
ENRICA-Seniors Study [13]	122	146	146	148	73	83	86	84	118	123	138	140	70	71	80	79
Aveiro Study [8, 19]	127	157	160	163	78	95	101	95	117	119	142	142	71	72	87	82

Values up to 0.5 were rounded to the lower unit and those greater than 0.5 to the higher unit

*ANRH* ambulatory nonresistant hypertension, *ARH* ambulatory (true) resistant hypertension, *CH* controlled hypertension, *DBP* diastolic blood pressure, *ENRICA* Study on Nutrition and Cardiovascular Risk in Spain, *JAMP* Japan Ambulatory Blood Pressure Monitoring Prospective, *NE* not evaluated, *SBP* systolic blood pressure, *WCURH* white coat uncontrolled resistant hypertension

**Fig. 2:**
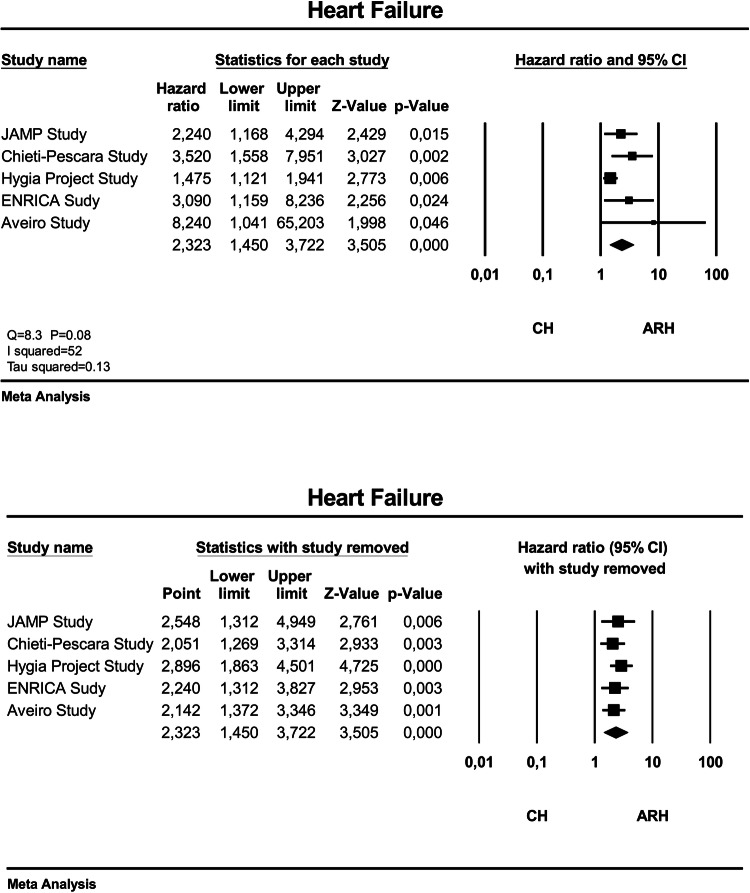
Forest plot showing the adjusted hazard ratio and 95% confidence interval (CI) between patients with ambulatory resistant hypertension (ARH) and those with controlled hypertension (CH) in the upper panel and sensitivity analysis (individual study removal) in the lower panel

Figure 2 (upper panel) gives the adjusted HR and 95% CI of the individual studies and of the overall analysis between ARH and CH. The overall adjusted HR was 2.32 (95% CI 1.45–3.72), *P* < 0.001, for ARH versus CH. The heterogeneity of the HR estimates across the studies was moderate (*I*^2^ = 52), though did not attain statistical significance (*P* = 0.08 for the Q statistic). We tried to explore potential sources of the heterogeneity by subgroup meta-analysis or meta-regression by considering characteristic of patients with ARH that were homogeneously reported across the studies, that is, follow-up length, age, sex, body mass index, diabetes, previous events, ambulatory BP, percentage of patients with events, and event rate. None of the above mentioned factors was significantly associated with heterogeneity. However, sensitivity analysis – individual study removal one by one (Fig. 2, lower panel) showed that none of the studies had a significant influential effect on the overall estimate.

**Fig. 3:**
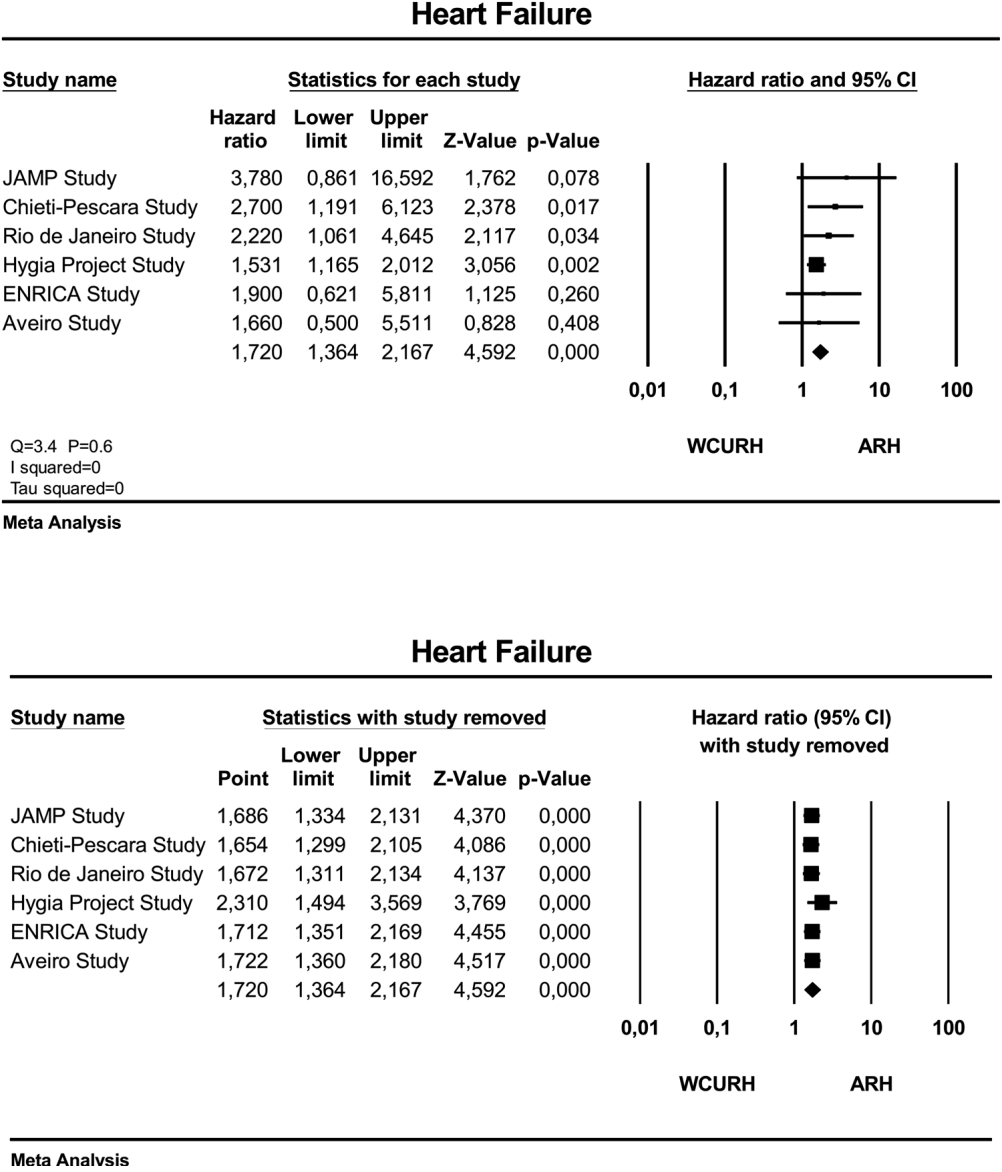
Forest plot showing the adjusted hazard ratio and 95% confidence interval (CI) between patients with ambulatory resistant hypertension (ARH) and those with white coat uncontrolled resistant hypertension (WCURH) in the upper panel and sensitivity analysis (individual study removal) in the lower panel

Figure 3 (upper panel) gives the adjusted HR and 95% CI of the individual studies and of the overall analysis between ARH and WCURH. The overall adjusted HR was 1.72 (95% CI 1.36–2.17), *P* < 0.001, for ARH versus WCURH. There was no heterogeneity across the studies (*I*^2^ = 0; *P* = 0.6 for Q statistic). Sensitivity analysis (Fig. 3, lower panel) showed that none of the studies had a significant influential effect on the overall estimate.

**Fig. 4:**
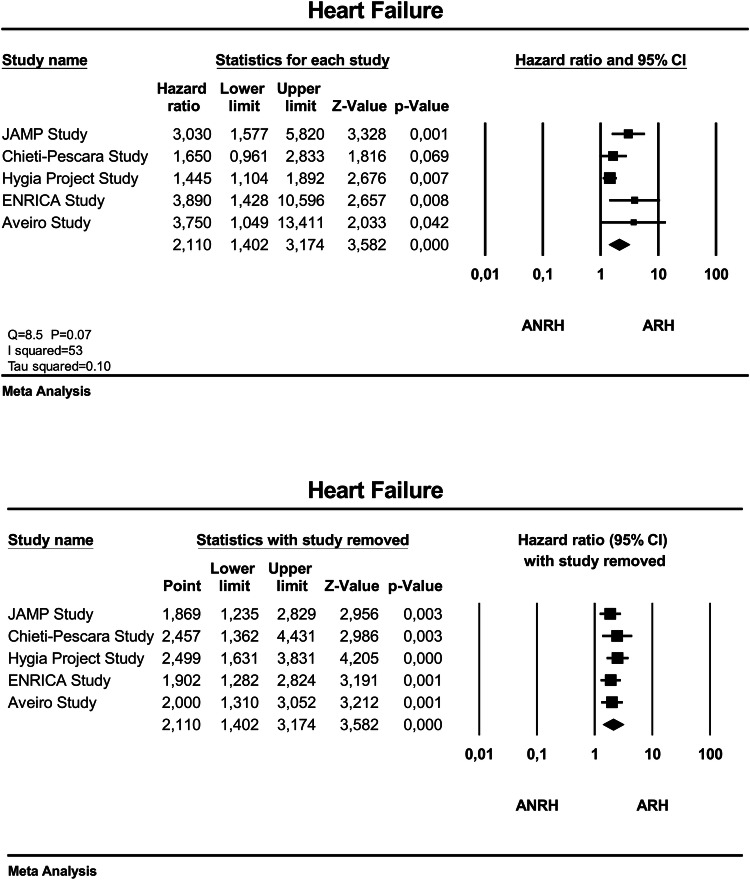
Forest plot showing the adjusted hazard ratio and 95% confidence interval (CI) between patients with ambulatory resistant hypertension (ARH) and those with ambulatory nonresistant hypertension (ANRH) in the upper panel and sensitivity analysis (individual study removal) in the lower panel

Figure 4 (upper panel) gives the adjusted HR and 95% CI of the individual studies and of the overall analysis between ARH and ANRH. The overall adjusted HR was 2.11 (95% CI 1.40–3.17), *P* < 0.001, for ARH versus ANRH. The heterogeneity of the HR estimates across the studies was moderate (*I*^2^ = 53), though did not attain statistical significance (*P* = 0.07 for the Q statistic). We tried to explore potential sources of the heterogeneity by subgroup meta-analysis or meta-regression by considering characteristic of patients with ARH that were homogeneously reported across the studies, as described in the comparison between ARH and CH. None of the above mentioned factors was significantly associated with heterogeneity. However, sensitivity analysis (Fig. 4, lower panel) showed that none of the studies had a significant influential effect on the overall estimate.

Generally, tests for funnel plot asymmetry are performed when ~10 studies are included in the meta-analysis, because when there are fewer studies the power of the tests is low to distinguish chance from real asymmetry. Thus, in the present study this analysis was not mandatory. However, for completeness, we explored for publication bias and small-study effect. In the comparison of ARH versus CH, ARH versus WCURH, and ARH versus ANRH, the Begg and Mazumdar test was always not significant, whereas Egger’s test was significant (*P* < 0.05) in the comparison of ARH versus CH and versus ANRH, and it approached significance (*P* = 0.06) in the comparison of ARH versus WCURH. When we applied Duval and Tweedie’s trim and fill method, in the comparison of ARH versus CH, ARH versus WCURH, and ARH versus ANRH, 2, 3, and 2 studies, respectively, appeared missing to the left side of the mean effect. In this context, the imputed point estimates were slightly lower (from 1.6 to 1.9) but remained significant (Supplementary Figure 1).

## Discussion

This meta-analysis shows that the risk of HF is significantly 1.7 to 2.3-fold higher in patients with ARH than in those with CH, WCURH, and ANRH.

Regarding some comparisons a moderate heterogeneity was found. By using subgroup meta-analysis or meta-regression we could not find variables that were able to explain the heterogeneity. However, sensitivity analyses demonstrated that none of the studies had a significant influential effect on the overall estimate. Moreover, when we evaluated the potential presence of publication bias and small-study effect and adjusted for missing studies by Duval and Tweedie’s trim and fill method, the risk estimates were slightly lower (from 1.6 to 1.9) but remained significant.

Concerning potential mechanisms explaining our findings, it should be remarked that 24-h systolic BP was more than 20 mmHg higher in patients with ARH than in those with CH and WCURH and tended to be higher than in those with ANRH, and 24-h diastolic BP was about 10 mmHg higher in patients with ARH than in those with CH and WCURH. Moreover, as previously reported, other factors observed in ARH such as fluid retention, activation of the sympathetic, and renin–angiotensin–aldosterone systems, a more severe vascular damage, and undetermined features could contribute to explain our findings [1, 40].

To the best of our knowledge, this is the first meta-analysis specifically evaluating the risk of HF in patients with ARH when compared to other ambulatory BP phenotypes. The prevalence of both ARH [1–4] and HF [22, 23] is progressively increasing in the population, due to various factors including aging, and their burden on public health is projected to increase over the years. Thus, a further effort should be done in patients with ARH, who are already taking three or more drugs, to reduce BP and risk of HF. In this context, it has recently been shown that a reduction of 5 mmHg of systolic BP is associated with a 13% reduction in HF risk [41].

Guidelines [1, 42–44] suggest adding mineraloreceptor blockers, mainly spironolactone to patients with resistant hypertension. It reduces clinic BP to a greater extent than other drugs [45], reduces ambulatory BP [46, 47], and improves outcome in patients with apparently resistant hypertension and HF [48]. However, other antihypertensive drug classes may also be added when needed [1].

A new drug class, that is, gliflozines might have potential in the treatment of ARH for reducing HF occurrence and BP. In randomized trials, gliflozines in primary prevention reduced HF hospitalization by about 30% in diabetic subjects of whom more than 90% also had hypertension and were already receiving antihypertensive drugs [26–29]. In these studies [26–29], more than 80% of patients received angiotensin converting enzyme inhibitors or angiotensin receptor blockers, more than 40% received diuretics, more than 50% received beta blockers, and in 2 of them [26, 29] more than 30% received calcium channel blockers. Systolic BP was further reduced by 3–4 mmHg in patients treated with gliflozines [26–29]. In the Empagliflozin Cardiovascular Outcome Event Trial in Type 2 Diabetes Mellitus Patients–Removing Excess Glucose Blood Pressure trial [30], empagliflozin reduced 24-h systolic BP (about 4 mmHg) irrespective of the number and type of antihypertensive drugs used. A further reduction of 24-h systolic BP (about 8 mmHg) was also observed in treated diabetic and hypertensive patients after adding empagliflozin in the SGLT2 inhibitor and Angiotensin Receptor Blocker Combination Therapy in Patients With Diabetes and Uncontrolled Nocturnal Hypertension study [31]. A preceding meta-analysis had shown that gliflozines are associated with a systolic BP reduction of about 4 mmHg when compared to placebo or active treatment [49]. Finally, in the Dapagliflozin Evaluation to Improve the Lives of Patients with Preserved Ejection Fraction Heart Failure trial, patients with apparently resistant hypertension showed the greatest reduction in the event rate with dapagliflozin (4.1/100 patient-years) when compared to non-resistant hypertension (2.7/100 patient-years) and controlled BP (0.8/100 patient-years) [50]. Thus, as gliflozines have a direct HF-suppressing effect (primary and secondary prevention) and further reduce BP load in individuals with/without diabetes and treated hypertension, this drug class appears a valuable tool to reduce HF burden in patients with ARH. This should be investigated in future trials.

### Study limitations

The present study has some limitations. First, few ethnic groups could be evaluated, and the results cannot be extrapolated to all ethnicities. Second, we assessed general/elderly treated hypertensive patients and our data cannot be extrapolated to other specific hypertensive populations, such as those with chronic kidney disease. Third, a similar set of covariates was used for adjustment in Cox multivariate analysis in each study but some of them included other covariates in the context of the specific study. Fourth, HF events were analyzed together without distinction between subtypes with preserved, mildly reduced, and reduced ejection fraction; in this context, future studies are needed to assess whether the impact of ARH on HF occurrence differs according to HF subtype. Our study also has some strengths. First, the same method, that is, ambulatory BP monitoring was used to detect out-of-office BP. Second, the same thresholds and criteria were used to define ARH and other ambulatory BP phenotypes. Third, a quite large sample size and number of HF events were included.

## Conclusions

This meta-analysis shows that treated hypertensive patients with ARH are at approximately twice the risk of developing HF than other ambulatory BP phenotypes. Therefore, every attempt should be done to identify this condition, which is increasing over time, and to find the best management for reducing HF occurrence. The addition to the established treatment strategy of new drugs that are successful in directly preventing HF and in reducing BP, such as gliflozines, may be a promising approach to mitigate the burden of HF in patients with ARH. In this context, future studies evaluating this therapeutic strategy should be performed.

### Supplementary information

Supplementary information

## Data Availability

The data that support the findings of the present study are available from the authors upon reasonable request.
