# Supplementary material for: Risk of heart failure in ambulatory resistant hypertension: a meta-analysis of observational studies
Source: Hypertens Res. 2024 Mar 14;47(5):1235–45. doi: 10.1038/s41440-024-01632-8 (PMC11073995; doi:10.1038/s41440-024-01632-8)
Supplement: Supplementary file 1 — Supplementary information [file 41440_2024_1632_MOESM1_ESM.doc]

**SUPPLEMENTARY INFORMATION**

**Risk of Heart Failure in Ambulatory Resistant Hypertension**

A Meta-Analysis of Observational studies

**Francesca Coccina^1^, Gil F. Salles^2^, José R. Banegas^3^, Ramón C. Hermida^4,5^, José M. Bastos^6^, Claudia R. L. Cardoso^2^, Guilherme C. Salles^7^, Mercedes Sánchez-Martínez^3,8^, Artemio Mojón^4,5^, José R. Fernández^4,5^, Carlos Costa^9^, Simão Carvalho^9^, Joao Faia^9^, Sante D. Pierdomenico^1^**

^1^Department of Innovative Technologies in Medicine & Dentistry, University “Gabriele d’Annunzio”, Chieti-Pescara, Chieti, Italy; ^2^Department of Internal Medicine, School of Medicine; Universidade Federal do Rio de Janeiro, Rio de Janeiro, Brazil; ^3^Department of Preventive Medicine and Public Health, Universidad Autónoma de Madrid and CIBERESP, Madrid, Spain; ^4^Bioengineering & Chronobiology Laboratories, Atlantic Research Center for Telecommunication Technologies (atlanTTic), Universidade de Vigo, Vigo, Spain. ^5^Bioengineering & Chronobiology Research Group, Galicia Sur Health Research Institute (IIS Galicia Sur), SERGAS-UVIGO, Vigo, Spain; ^6^School of Health Sciences and Institute of Biomedicine-iBiMED, University of Aveiro, Aveiro, Portugal; ^7^Deparment of Civil Engineering, Polytechnic School, Universidade Federal do Rio de Janeiro, Rio de Janeiro, Brazil; ^8^Department of Health Science, Universidad Católica Santa Teresa de Jesús de Ávila, Ávila, Spain; ^9^Cardology Department of Centro Hospitalar Baixo Vouga, Aveiro, Portugal.

Corresponding author: Sante D. Pierdomenico, MD - Tel: +39-3299286871 - e-mail: [sante.pierdomenico@unich.it](mailto:sante.pierdomenico@unich.it)

Supplementary Information: Supplementary Tables 1-3 and Supplementary Figure 1.

**Supplementary Table 1.** Definition of heart failure in selected studies

JAMP Study^20^ An event requiring hospital admission due to clinical manifestations of HF such as the presence of dyspnea, systemic edema or

edema in lower limbs, third heart sound, pulmonary congestion or cardiac dilation on chest X-ray, and requirement for treatment

Chieti-Pescara Study^21^ HF requiring hospitalization; diagnosis of HF was based on symptoms and signs of HF, BNP or NT-proBNP when available,

chest X-ray and echocardiographic examination (HF subtypes were reclassified as HFrEF, HFmrEF, HFpEF)

Rio de Janeiro Study^16^ Hospitalization for symptoms and signs of HF and an objective demonstration of HFrEF

Hygia Project Study^15^ Hospitalization or emergency unit visit for a clinical syndrome that presents with multiple signs and symptoms consistent with cardiac

decompensation or inadequate cardiac pump function when appearing during follow-up in participants without previous HF symptoms

ENRICA-Seniors Study^13^ Patients were considered to suffer from HF if their respective diagnoses or their related ICPC-2 or ICD-9 codes were registered in their

medical records, considering the following definitions and diagnostic criteria: HF (ICPC-2: K77. ICD-9: 428)

Aveiro Study^8,19^ Clinical syndrome consisting of symptoms and signs of HF requiring hospitalisation

BNP, B-type natriuretic peptide; ENRICA, Study on Nutrition and Cardiovascular Risk in Spain; JAMP, Japan Ambulatory Blood Pressure Monitoring Prospective; HF, heart failure; HFmrEF, heart failure with mild reduced ejection fraction (40-49%); HFpEF, heart failure with preserved ejection fraction (> 50%); HFrEF, heart failure with reduced ejection fraction (< 40%); NT-proBNP, N-terminal pro-BNP. Symptoms, signs and chest X-ray findings of heart failure are those previously described [22,23].

**Supplementary Table 2.** Other characteristics of selected studies

Study Population Ethnicity Covariates in the Multivariate analysis

JAMP Study^20^ Treated hypertensive patients Japanese Age, sex, BMI, diabetes, previous events

Chieti-Pescara Study^21^ Elderly treated hypertensive patients Caucasian Age, diabetes, LVH, ALVSD, LAE, eGFR, PEMS, nondipping, previous events

Rio de Janeiro Study^16^ Treated hypertensive patients* Mixed Latinos Age, sex, BMI, diabetes, smoke, TC, HDL, eGFR, drug number, previous events

Hygia Project Study^15^ Treated hypertensive patients Caucasian Age, sex, waist perimeter, eGFR, chronic kidney disease, previous events

ENRICA-Seniors Study^13^ Elderly treated hypertensive patients Caucasian Age, BMI, diabetes, previous events

Aveiro Study^8,19^ Treated hypertensive patients Caucasian Age, sex, BMI, dyslipidaemia, previous events

ALVSD; asymptomatic left ventricular dysfunction; BMI, body mass index; eGFR, estimated glomerular filtration rate; ENRICA, Study on Nutrition and Cardiovascular Risk in Spain; HDL, high density lipoprotein cholesterol; JAMP, Japan Ambulatory Blood Pressure Monitoring Prospective; LAE, left atrial enlargement; LVH, left ventricular hypertrophy; PEMS, pre-awake morning surge of blood pressure; TC, total cholesterol. *Apparent resistant hypertension.

**Supplementary Table 3**. Assessment of quality of studies according to the Newcastle-Ottawa Scale

| Study | Representativeness of the exposed cohort | Selection of the non exposed cohort | Ascertainment of exposure | Demonstration that outcome of interest was not present at start of study | Comparability of cohorts on the basis of design or analysis | Assessment of outcome | Was follow-up long enough for outcomes to occur? | Adequacy of follow-up of cohorts | Total score |
| --- | --- | --- | --- | --- | --- | --- | --- | --- | --- |
| JAMP Study^20^ | * | * | * | * | ** | * | * | * | 9 |
| Chieti-Pescara Study^21^ | * | * | * | * | ** | * | * | * | 9 |
| Rio de Janeiro Study^16^ | * | * | * | * | ** | * | * | * | 9 |
| Hygia Project Study^15^ | * | * | * | * | ** | * | * | * | 9 |
| ENRICA-Seniors Study^13^ | * | * | * | * | ** | * | * | * | 9 |
| Aveiro  Study^8,19^ | * | * | * | * | ** | * | * | * | 9 |

ENRICA, Study on Nutrition and Cardiovascular Risk in Spain; JAMP; Japan Ambulatory Blood Pressure Monitoring Prospective.


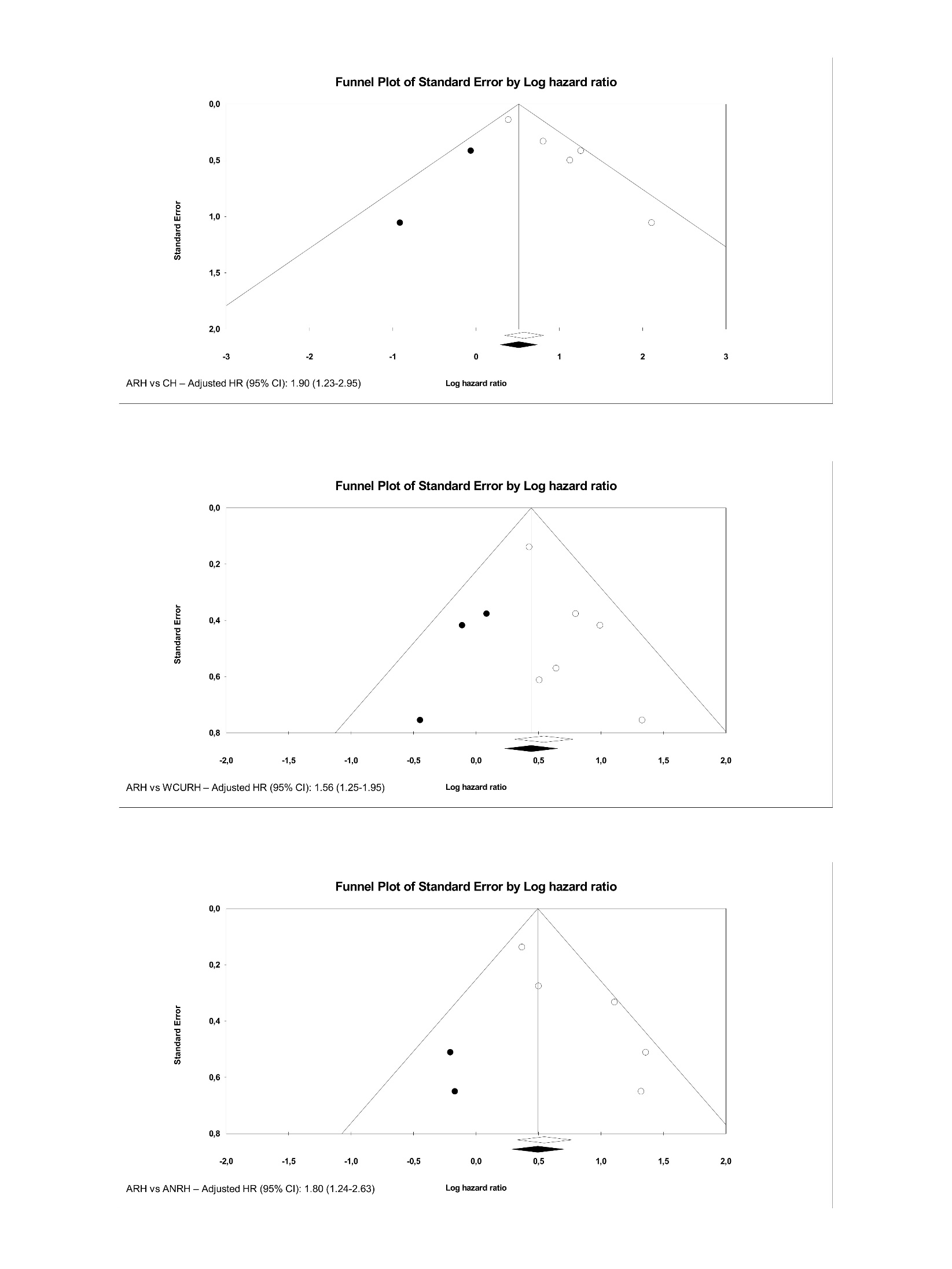


**Supplementary Figure 1**. Funnel plots. The empty circles correspond to the studies included in the meta‐analyses and the open diamond represents the point estimate, with lower and upper limits, under the random effects model; the solid black circle corresponds to the imputed missing studies and the solid black diamond represents the imputed point estimate, with lower and upper limits, after use of Duval and Tweedie’s trim-and-fill method. ANRH, ambulatory nonresistant hypertension; ARH, ambulatory resistant hypertension; CH, controlled hypertension; CI, confidence interval; HR, hazard ratio; WCURH, white coat uncontrolled resistant hypertension.
